# Serological Response in RT-PCR Confirmed H1N1-2009 Influenza A by Hemagglutination Inhibition and Virus Neutralization Assays: An Observational Study

**DOI:** 10.1371/journal.pone.0012474

**Published:** 2010-08-30

**Authors:** Mark I. Chen, Ian G. Barr, Gerald C. H. Koh, Vernon J. Lee, Caroline P. S. Lee, Robert Shaw, Cui Lin, Jonathan Yap, Alex R. Cook, Boon Huan Tan, Jin Phang Loh, Timothy Barkham, Vincent T. K. Chow, Raymond T. P. Lin, Yee-Sin Leo

**Affiliations:** 1 Clinical Epidemiology, Tan Tock Seng Hospital, Singapore, Singapore; 2 Epidemiology and Public Health, National University of Singapore, Singapore, Singapore; 3 Duke-NUS Graduate Medical School, Singapore, Singapore; 4 World Health Organization Collaborating Centre for Reference and Research on Influenza, Melbourne, Victoria, Australia; 5 Centre for Health Services Research, National University of Singapore, Singapore, Singapore; 6 Biodefence Centre, Ministry of Defence, Singapore, Singapore; 7 Centre for Epidemiology and Population Health, Australian National University, Canberra, Australian Capital Territory, Australia; 8 Department of Infectious Diseases, Tan Tock Seng Hospital, Singapore, Singapore; 9 National Public Health Laboratory, Ministry of Health, Singapore, Singapore; 10 Department of Statistics and Applied Probability, National University of Singapore, Singapore, Singapore; 11 Detection and Diagnostics Laboratory, DSO National Laboratories, Singapore, Singapore; 12 Department of Laboratory Medicine, Tan Tock Seng Hospital, Singapore, Singapore; 13 Department of Microbiology, National University of Singapore, Singapore, Singapore; University of Georgia, United States of America

## Abstract

**Background:**

We describe the serological response following H1N1-2009 influenza A infections confirmed by reverse-transcriptase polymerase chain reaction (RT-PCR).

**Methodology and Principal Findings:**

The study included patients admitted to hospital, subjects of a seroepidemiologic cohort study, and participants identified from outbreak studies in Singapore. Baseline (first available blood sample) and follow-up blood samples were analyzed for antibody titers to H1N1-2009 and recently circulating seasonal influenza A virus strains by hemagglutination inhibition (HI) and virus micro-neutralization (VM) assays. 267 samples from 118 cases of H1N1-2009 were analyzed. Geometric mean titers by HI peaked at 123 (95% confidence interval, CI 43-356) between days 30 to 39. The chance of observing seroconversion (four-fold or greater increase of antibodies) was maximized when restricting analysis to 45 participants with baseline sera collected within 5 days of onset and follow-up sera collected 15 or more days after onset; for these participants, 82% and 89% seroconverted to A/California/7/2009 H1N1 by HI and VM respectively. A four-fold or greater increase in cross-reactive antibody titers to seasonal A/Brisbane/59/2007 H1N1, A/Brisbane/10/2007 H3N2 and A/Wisconsin/15/2009 H3N2 occurred in 20%, 18% and 16% of participants respectively.

**Conclusions and Significance:**

Appropriately timed paired serology detects 80–90% RT-PCR confirmed H1N1-2009; Antibodies from infection with H1N1-2009 cross-reacted with seasonal influenza viruses.

## Introduction

The novel influenza A (H1N1-2009) virus first identified in April 2009 in the United States (US) and Mexico spread rapidly across the world,[1], [2], [3] with Singapore experiencing its first wave of infections from June to September 2009.[4] In Singapore and elsewhere, serological surveys, using either hemagglutination inhibition or virus neutralization, have been used to assess the extent of H1N1-2009 infections.[5], [6], [7], [8], [9] Serological assays have also been used to detect antibody responses against H1N1-2009 in vaccine efficacy studies.[10], [11], [12]

Although hemagglutination inhibition assays have been widely used to diagnose seasonal influenza and assess response to seasonal influenza vaccines,[13], [14] data is still needed to assess the performance of such assays for pandemic H1N1-2009, the timing of the serological response and the proportion of H1N1-2009 cases which seroconvert. Recent work by Miller et al suggests that detectable antibodies largely arise between 8 to 14 days after onset, with more than 85% of subjects tested having antibody titers of 32 or greater by hemagglutination inhibition after 15 days.[7] Some data has also been published on the sensitivity of paired serology by hemagglutination inhibition and virus neutralization for diagnosis of H1N1-2009, but the study involved a small number of confirmed cases and did not take into account how the assay might be affected by the timing of baseline and follow-up sample collection.[15] In addition, there is also little data at present on the extent to which cross-reactive antibodies to other influenza A strains develop following pandemic H1N1-2009 infection.

This study therefore aims to address the above knowledge gaps by profiling the serological responses in a cohort of individuals with naturally acquired H1N1-2009 infection confirmed by reverse-transcriptase polymerase chain reaction (RT-PCR).

## Methods

### Objectives

We conducted an observational study to determine the optimal timing of baseline and follow-up sample collection in a set of RT-PCR-confirmed cases of pandemic H1N1-2009 influenza A infections, estimate the sensitivity of paired serology by hemagglutination inhibition assays in detecting such cases while accounting for the timing of paired samples, compare results obtained with hemagglutination inhibition with those from virus microneutralization assays, and assess if cross-reactive antibodies to other influenza strains developed following infection with H1N1-2009.

### Participants

The study involved the collection of one or more blood samples for serology from consenting participants in Singapore. For each participant, we refer to the first blood sample as the “baseline” sample (even if collected after onset of illness), and all subsequent samples as “follow-up” samples.

Participants enrolled in this study were drawn from 3 sources. The first was from an observational study of patients admitted to Tan Tock Seng Hospital (TTSH), Singapore. TTSH was the designated facility for isolation and treatment of adult patients with RT-PCR confirmed H1N1-2009 infection during the containment phase of the Singapore epidemic.[16] Cases admitted to TTSH were invited to participate in a clinical study to characterize the infection. Consenting participants had a baseline blood sample collected on the day of enrolment, and follow-up samples obtained every other day thereafter during their admission. Following discharge, participants were requested to return for follow-up samples at 2–4 weeks and 6–8 weeks after the enrolment date. The second source was from military personnel and hospital staff who were part of a sero-incidence cohort study of pandemic H1N1-2009 incidence.[17], [18] Participants were enrolled before there was widespread community transmission in Singapore and therefore contributed their baseline blood samples prior to infection. Up to two additional blood samples were collected during the first epidemic wave. Participants who had RT-PCR confirmed H1N1-2009 influenza A infection were included. The final source was from participants identified during outbreak investigations in two military camps (15 participants) and one long-term care facility (8 participants) who had had H1N1-2009 influenza A detected by RT-PCR as well as serial blood samples which were collected in the course of the investigations.

Participants also contributed information on demographics, past medical history, influenza related symptoms, date of illness onset, and details on oseltamivir treatment.

### Laboratory confirmation of H1N1 infection by RT-PCR

For the diagnosis of influenza, nasal and throat samples obtained with flocked swabs were transported in universal transport medium (Copan) either to the Department of Laboratory Medicine at TTSH or the DSO National Laboratories. Probe-based RT-PCR was conducted with in-house or US Centers for Disease Control and Prevention methods. [19]

### Serological assays by hemagglutination inhibition and virus neutralization

Both hemagglutination inhibition (HI) and virus microneutralization (VM) assays were performed by the World Health Organization Collaborating Centre for Reference and Research on Influenza in Melbourne, Australia.

Details on the HI assays have been published elsewhere.[20] These followed standard protocols with sera titrated in two-fold dilutions from 1∶10 to 1∶1280 and tested against the A/California/7/2009 (H1N1) pandemic virus. To investigate potential cross-reaction by HI assay, a subset of samples was also tested against three other influenza A strains. These were A/Brisbane/59/2007 H1N1 and A/Brisbane/10/2007 H3N2 (components of the 2009 Northern hemisphere seasonal influenza vaccine), and A/Wisconsin/15/2009 H3N2 (an antigenic drift variant of H3N2 similar to those circulating in Singapore in 2009 and which has since been recommended as the updated H3N2 component to be included in the trivalent seasonal influenza vaccines for the Southern Hemisphere 2010 and Northern Hemisphere 2010-11 influenza seasons).[21]

Virus microneutralization assays were performed on samples with sufficient volume. Undiluted sera were inactivated by incubation at 56°C for 30 min. Equal volumes of heat-treated serum (at two-fold dilutions beginning at 1∶10) and 100 tissue culture infective dose (TCID)_50_ of the egg-propagated wild-type A/California/7/2009 virus were mixed and incubated in duplicate at 35°C for 1 hour. The virus-serum mix was then added to washed, near-confluent (90%) monolayers of Madin-Darby canine kidney cells (CCL-34, ATCC) in 96-well flat-bottomed plates (Greiner Bio One) and incubated at 35°C with 5% CO_2_ for 2 h. The virus/serum mix was replaced with fetal-calf-serum-free culture medium supplemented with 4 µg/ml trypsin (Sigma) and the cells were incubated at 35°C with 5% CO_2_. Four days later, supernatant from each well was assayed for virus by a hemagglutination assay with 1% turkey erythrocytes. Each titer was expressed as the reciprocal of the highest dilution of serum where hemagglutination was prevented. Positive and negative control human and animal sera as well as no serum and no virus controls were included in each assay.

### Ethics

Written informed consent was obtained from all participants. The study was approved by the ethics review boards of the National Healthcare Group, Singapore Armed Forces, National University of Singapore and Australian National University.

### Data analysis and statistical methods

Date of illness onset was based on the earliest reported symptoms, except for 3 participants (2 sero-incidence cohort participants and 1 outbreak case) without clear symptom onset dates where the date of the positive RT-PCR test was used instead. We then computed the time from illness onset to date of blood sample collection to the nearest day. Seroconversion was defined as a 4-fold or greater increase in antibody titer between the baseline sample and follow-up blood sample for the same individual. For participants with more than one follow-up sample, the sample with the highest titer amongst all follow-up samples for that participant was used to assess if seroconversion occurred. In situations where the highest titer observed for a given participant was found to occur in several follow-up samples for that participant, the date of the latest follow-up sample was used when investigating the effect of time from illness onset to follow-up sample.

Geometric mean titers (GMTs) were computed by assigning a titer value of 5 for samples where antibodies were undetectable (<10). In analyses where some individuals contributed more than one observation, robust standard errors were used to construct 95% confidence intervals (CIs). Statistical testing was performed with the level of significance set at p<0.05. For proportions, chi-squared tests were used; for testing the relative change between baseline and follow-up sample titers (i.e. the latter titer divided by the former), we used the Wilcoxon rank-sum and Kruskal-Wallis tests for binary and multichotomous variables respectively; for comparing baseline and follow-up titers to different influenza strains, the Wilcoxon signed-rank test for paired data was used. All data was analyzed using STATA 10.0 (StataCorp, College Park, Texas).

## Results

### Profile of study participants

Table 1 gives the characteristics of all 118 individuals included in our study. The majority (57%, 67/118) were cases admitted to TTSH, followed by participants from the sero-incidence cohort study (24%, 28/118), and cases identified during outbreak investigations (19%, 23/118). Since 25 of 28 participants from the sero-incidence cohort and 15 of the 23 outbreak-related cases were military personnel, there was a predominance of young males in our study, with 69% (81/118) aged less than 35 years, and 71% (84/118) being male. Asthma or bronchitis was reported by 12% of all participants (14/118), with only a small number reporting other co-morbid conditions. Most of the participants received oseltamivir treatment (76%, 90/118), with 51% (46/90) receiving treatment within 2 days or less of onset. There were a total of 267 blood samples from the 118 individuals (median of 2 per participant). Due to incomplete follow-up, not all participants provided complete sets of scheduled blood samples, but pair-wise assessment of change in titers was possible in 90 (76%) participants who contributed samples at two or more time points. A more detailed breakdown on the contribution of hospitalized cases, sero-incidence cohort participants and outbreak cases to the number of individuals and samples analyzed is given in Table 2. Hospitalized cases were less likely to have paired samples than sero-incidence cohort participants and outbreak cases, but still accounted for the majority of participants (50%, 45/90) with paired serology for assessment.

**Table 1:** Characteristics of study participants (N = 118).

Participant characteristics		
Source	Hospitalized cases	67 (57)
	Seroincidence cohort	28 (24)
	Outbreak cases	23 (19)
Age distribution in years	Median (range)	25 (19–62)
	<20 years	8 (7)
	20–34 years	73 (62)
	35–49 years	19 (16)
	≥50 years	18 (15)
Gender	Male	84 (71)
	Female	34 (29)
Co-morbid conditions	Asthma or bronchitis	14 (12)
	Hypertension or dyslipidemia	9 (8)
	Cardiovascular disease	2 (2)
	Diabetes mellitus	4 (3)
	Cancer and immunological disorders*	8 (7)
Oseltamivir treatment	Yes	90 (76)
	No	23 (19)
	Unknown	5 (4)
Onset to oseltamivir treatment	Timing unknown	9 (10)†
	2 days or less	46 (51)†
	3 or more days	35 (39)†
Number of samples	Median (range)	2 (1–7)
	One	28 (24)
	Two	46 (39)
	Three or more	44 (37)

NB: Unless otherwise stated, data presented are number of participants with percentages in brackets.

*Hodgkin's lymphoma in remission, leukemia in remission, breast cancer on tamoxifen, human immunodeficiency virus infection on antiviral treatment, sarcoidosis, rheumatoid arthritis, systemic lupus erythematosus, long-term prednisolone therapy for unspecified endocrine disorder.

† As % of subjects who received oseltamivir treatment.

**Table 2:** Detailed breakdown on sources of study participants and samples.

		Hospitalized cases	Sero-incidence cohort	Outbreak cases	Total
All participants	No. of participants	67 (57)	28† (24)	23‡ (19)	118 (100)
	No. of samples	148 (55)	76 (28)	43 (16)	267 (100)
Two or more samples	No. of participants	45 (50)	28 (31)	17 (19)	90 (100)
	No. of samples	126 (53)	76 (32)	37 (15)	239 (100)

NB: Numbers in brackets are row percentages.

† 25 military personnel and 3 hospital staff from Tan Tock Seng Hospital.

‡ 15 participants from military outbreaks and 8 from the long-term care facility outbreak.

### Timing of serological response by hemagglutination inhibition assays

**Figure 1:**
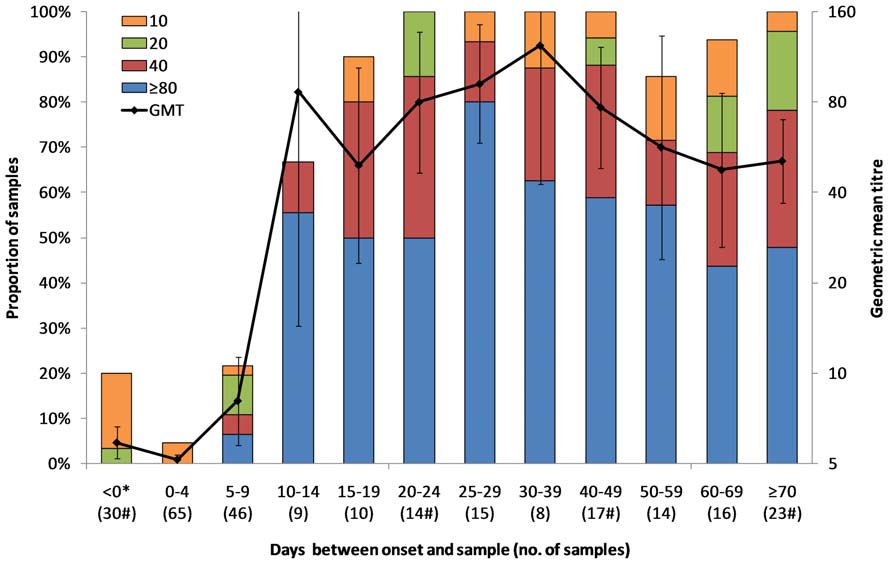
Hemagglutination inhibition titers by time from illness onset to sample collection. Includes 267 samples from 118 individuals. Samples taken before onset in sero-incidence cohort participants are grouped (as <0* days), as are those taken after 70 days (≥70 days), with other observations summarized in 5 day intervals up to 29 days, 10 day intervals from 30 to 69 days. The number of samples in each interval is in brackets; intervals marked with # include 7 samples from the 3 individuals whose date of positive PCR test was used instead of onset dates: <0 (2 samples), 20 to 24 (1 sample), 40 to 49 (2 samples) and ≥70 (2 samples). Colored stacked bars give the proportion with titers of 10, 20, 40 and ≥80 while the line denotes the geometric mean titer with error bars depicting 95% confidence intervals. The upper limit is off the scale for days 10–14 (518) and days 30–39 (356).

Figure 1 shows antibody titers by time between onset and sample collection, based on HI assays for all 267 samples. Although antibodies could be detected in 6 of the 30 samples (20%) collected before illness onset, none had titers of 40 or greater. Within the first 14 days, the proportion with titers of 40 or greater increased rapidly from 0% (0/65) on days 0–4, to 11% (5/46) on days 5–9, 67% (6/9) on days 10–14, and 80% (8/10) on days 15–19, with a corresponding increase in the geometric mean titer. Beyond day 15, the increase in titers was more gradual; the proportion with titers of 40 or greater peaked at 93% (14/15) between days 25–29, and GMT was highest in samples collected between days 30–39 (123, 95% CI 43 to 356).

**Figure 2:**
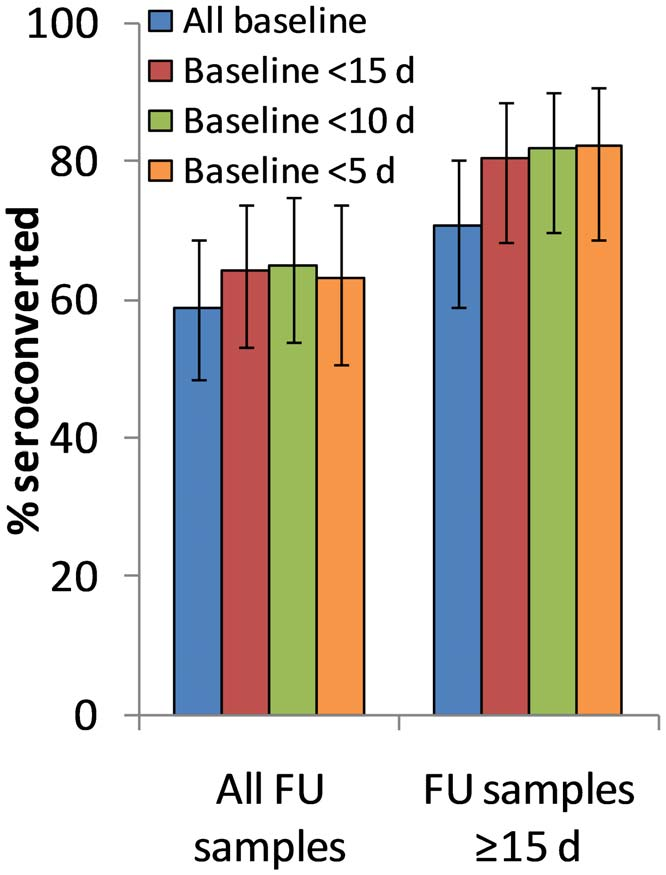
Seroconversion on hemagglutination inhibition assay, by timing of sample collection. Titers between baseline and follow-up (FU) sample is compared, for all participants regardless of time from onset to follow-up sample (n = 90), and restricting to participants whose follow-up sample was taken 15 or more days after onset (n = 68), with error bars depicting 95% confidence intervals; columns of different colors depict different cut-off points for time from onset to baseline sample. Seroconversion was defined here as a four-fold or greater increase in antibody titer.

Overall, seroconversion was observed in 53 of the 90 participants with two or more samples (59%, 95% CI 49% to 68%). Seroconversion percentages varied by the timing of the baseline and follow-up samples relative to illness onset (Figure 2). The chance of observing seroconversion was maximized at 82% (95% CI 69% to 91%) when the analysis was restricted to the 45 participants where the baseline sample was collected earlier (before onset or less than 5 days from illness onset), and the follow-up sample collected later (15 days or more days after illness onset). To reduce potential confounding by time of sample collection, we restricted to this subset of 45 participants when analyzing for associations between seroconversion and other participant characteristics.

### Comparison of hemagglutination inhibition and virus microneutralization

**Figure 3:**
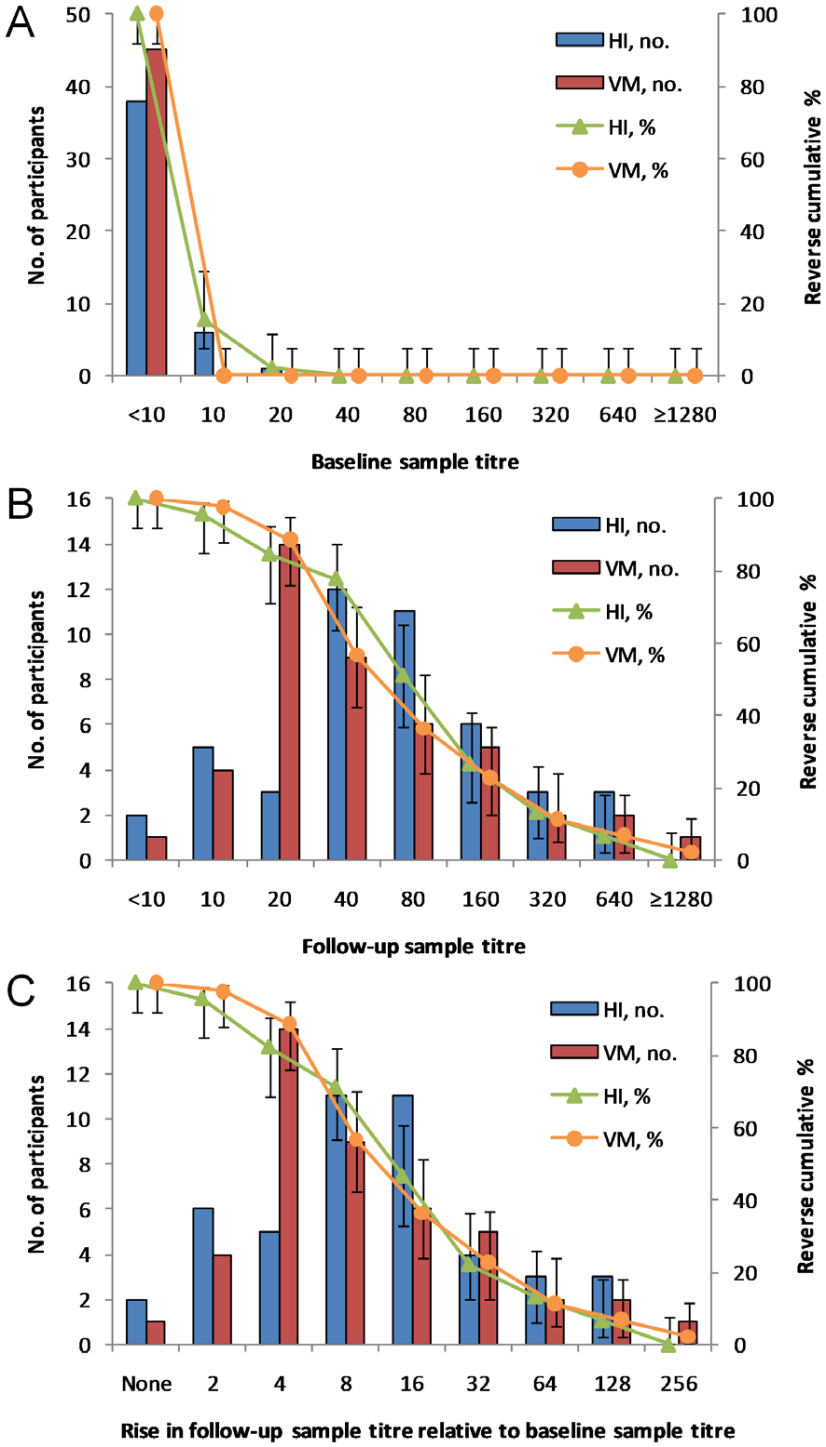
Comparison between hemagglutination inhibition (HI) and virus neutralization (VM). A) baseline sample titers (n = 45 for HI, n = 45 for VM); B) follow-up sample titers (n = 45 for HI, n = 44 for VM); C) fold rise in titer for follow-up relative to baseline titer (n = 45 for HI, n = 44 for VM). In all three panels, lines denote the reverse cumulative distribution with error bars representing the 95% confidence intervals.

Figure 3A compares baseline titers obtained using HI and VM assays, restricted again to the 45 participants with optimally timed baseline and follow-up samples. No detectable antibodies (titers<10) were observed in 84% (38/45) by HI assay compared to 100% (45/45) by VM. Figure 3B compares follow-up sample titers in 45 participants by HI, and in 44 participants by VM assays (one sample was not tested by VM due to insufficient sera). While the distribution derived from the two tests was fairly similar, follow-up GMTs were 59 (95% CI 41-85) by HI compared to 47 (95% CI 32-68) by VM assay, the difference being of borderline significance (p = 0.06 by Wilcoxon signed-rank test on 44 observations). Figure 3C shows the effect of using different cut-off points for defining seroconversion. Almost all participants had some detectable increase in titers, with 96% (43/45) having a two-fold or greater increase in titer by HI compared to 98% (43/44) by VM. When using the traditionally accepted cut-off point of a four-fold or greater increase in titers, 82% (37/45) were classified as having seroconverted by HI, which was slightly less than the 89% (39/44) by the VM assay. However, because a greater proportion of participants had follow-up titers of 40 or greater by HI than by VM, 71% (32/45) had an 8-fold or greater increase in titers by HI compared with only 57% (25/44) by VM.

We also investigated if the probability of observing seroconversion on HI or VM assays was associated with various participant characteristics. Notably, seroconversion was not significantly associated with treatment or timing of treatment with oseltamivir, nor with age, gender, co-morbidities and baseline antibody titers; the relative change between baseline and follow-up titers were also not affected by any of these participant characteristics.

### Cross-reactive antibodies detected on hemagglutination inhibition assays

Investigation of cross-reactive serological response on hemagglutination inhibition was also restricted to the same subset of 45 participants with optimally timed baseline and follow-up samples. Table 3 shows titers in baseline and follow-up samples to pandemic H1N1-2009 (A/California/7/2009 H1N1) and three seasonal influenza A strains. None of the participants had titers of 40 or greater to A/California/7/2009 H1N1 in their baseline samples, whereas 42% (19/45), 24% (11/45) and 18% (8/45) exhibited titers of 40 or greater to A/Brisbane/59/2007 H1N1, A/Brisbane/10/2007 H3N2 and A/Wisconsin/15/2009 H3N2 respectively. GMT in the baseline sample was highest for A/Brisbane/59/2007 H1N1 (18, 95% CI 12-26), but lowest for A/California/7/2009 H1N1 (6, 95% CI 5-6). GMTs to all four strains increased in follow-up samples, but the increase was statistically significant only for A/California/7/2009 H1N1 and A/Brisbane/59/2007 H1N1 (p<0.01 for both). Interestingly, seroconversion to A/Brisbane/59/2007 H1N1, A/Brisbane/10/2007 H3N2 and A/Wisconsin/15/2009 H3N2 occurred in 9 (20%), 8 (18%) and 7 (16%) participants, all of whom also seroconverted to A/California/7/2009 H1N1.

**Table 3:** Hemagglutination inhibition titers and seroconversion to different influenza A strains (N = 45).

		Antibody titers, %					
Strain	Sample	<10	10–20	≥40	GMT (95% CI)	p-value*	SC†, % (95% CI)
A/California/7/2009 H1N1 (pandemic)	Baseline	84	16	0	6 (5–6)	-	-
	Follow-up	4	18	78	59 (41–85)	<0.01	82 (69–91)
A/Brisbane/59/2007 H1N1 (seasonal)	Baseline	40	18	42	18 (12–26)	-	-
	Follow-up	29	11	60	36 (23–59)	<0.01	20 (11–34)
A/Brisbane/10/2007 H3N2 (seasonal)	Baseline	40	36	24	15 (10–22)	-	-
	Follow-up	36	24	40	20 (13–30)	0.80	18 (9–31)
A/Wisconsin/15/2009 H3N2 (seasonal)	Baseline	67	16	18	10 (7–14)	-	-
	Follow-up	60	7	33	13 (9–20)	0.43	16 (8–29)

*Wilcoxon signed-rank test comparing baseline and follow-up titers.

† SC: seroconversion (4-fold or greater increase in titer).

## Discussion

There is currently limited data on the antibody response following H1N1-2009 infection as detected on serological assays. In our study, antibody titers increased rapidly in the first 2 weeks, and collection of acute samples less than 5 days from illness onset, and convalescent samples more than 2 weeks after illness onset, maximized the proportion of RT-PCR confirmed infections which seroconverted on hemagglutination inhibition assays. In addition, we showed that more than 80% of RT-PCR confirmed H1N1-2009 cases seroconvert using hemagglutination inhibition and virus microneutralization assays, and demonstrated the development of cross-reactive antibodies to other influenza A strains following H1N1-2009 infection. These key characteristics of the antibody response have implications on the interpretation of serological assays for pandemic H1N1-2009.

Our finding, on the rapid increase of detectable antibodies by hemagglutination inhibition in the first two weeks, is fairly similar to what Miller and colleagues observed.[7] In addition to what they found, we were able to demonstrate how the timing of the blood sampling with respect to symptom onset affects the characteristics of paired serological assays. To optimize assay performance, we found that convalescent sera should be collected at least 2 weeks after illness onset. Testing samples collected less than 5 days after onset of illness maximized the proportion observed to seroconvert, although the cut-off point for acute sera was less critical. We also demonstrated that, with appropriately timed samples, 82% and 89% of the RT-PCR confirmed cases seroconverted on the HI and VM assays respectively. These results are fairly close to those described by Cowling et al, who reported that slightly less 80% and more than 95% of 19 RT-PCR confirmed pandemic H1N1-2009 cases with paired serology had four-fold or greater increase in titers on HI and VM assays respectively.[15] However, unlike Cowling who suggested that subjects given oseltamivir treatment early in the course of their disease might have a diminished convalescent antibody response, we did not find this to be so for oseltamivir use, nor for any of the participant characteristics investigated. It must be noted, however, that our study was under-powered to investigate key features of importance, such as co-morbid conditions which might depress immune function since such subjects were under-represented in our study.

Finally, we also documented the development of cross-reactive antibodies to other strains of influenza A following RT-PCR confirmed H1N1-2009 infection. While we cannot rule out the possibility that recent H3N2 or seasonal H1N1 infections preceding the episode of H1N1-2009 infection might have accounted for some of our findings, we note that there was little circulation of seasonal H1N1viruses throughout the study period, and H3N2 activity had also largely waned by the time our H1N1-2009 cases were sampled.[22] Heterotypic cross-reactive antibodies following suspected infection with pandemic influenza strains have been previously reported. For instance, one study on the 1968 influenza pandemic in Singapore reported that some patients with clinically diagnosed influenza who seroconverted to A/Singapore/1/68 (H3N2), the pandemic strain circulating in Singapore then, also seroconverted to A/Singapore/1/57 (H2N2), the causative strain of the previous pandemic about a decade earlier.[23] It is unclear as to the extent these cross-reactive antibodies are protective to the respective influenza A strains in vivo, but such antibodies could be one pathway for the heterotypic protection observed in an epidemiological study which showed how adults previously infected with H1N1 influenza in the years prior to the 1957 pandemic were protected during the 1957 H2N2 pandemic.[24] Our observations on cross-reactive antibodies also have implications for interpreting data from serologic surveys which assess relative infection rates of H3N2 and H1N1-2009 while the two strains continue to co-circulate.

### Limitations

Our study was based on participants aggregated from three separate sources which differed in their population characteristics and timing of sample collection. Due to low follow-up rates for the 2 to 4 week and 6 to 8 week samples, hospitalized cases were overrepresented in earlier samples and underrepresented in later samples. We suggest that the main bias that would arise from aggregating such subjects for analysis relates to the differences in the timing of baseline and follow-up samples for the three different groups, and we hence attempted to reduce such biases by restricting analyses to participants with baseline and follow-up sera collected within an appropriate time-frame. However, in doing so, the study sample size available for detecting differences in serological response by participant characteristics was reduced.

### Conclusions

In spite of the above limitations, our study provides important information profiling key aspects of the serological response following infection with the pandemic H1N1-2009 strain. Our findings also have implications for the serological diagnosis of H1N1-2009 based on paired serum samples, as well as the conduct of future serological surveys for influenza. Further studies are needed to understand the significance and mechanisms of the cross-reactive antibody responses to other influenza strains that occur in some individuals following H1N1-2009 infection.
